# “Look at the Whole Me”: A Mixed-Methods Examination of Black Infant Mortality in the US through Women’s Lived Experiences and Community Context

**DOI:** 10.3390/ijerph14070727

**Published:** 2017-07-05

**Authors:** Maeve E. Wallace, Carmen Green, Lisa Richardson, Katherine Theall, Joia Crear-Perry

**Affiliations:** 1 Mary Amelia Women’s Community Health Education Center, Department of Global Community Health and Behavioral Sciences, Tulane University School of Public Health and Tropical Medicine, 1440 Canal St., Suite 2210, New Orleans, LA 70112, USA; ktheall@tulane.edu; 2 National Birth Equity Collaborative, 4747 Earhart Blvd, Suite I, New Orleans, LA 70125, USA; carmen.nbec@gmail.com (C.G.); lrichrds@gmail.com (L.R.); drjoia.nbec@gmail.com (J.C.-P.); 3 Institute of Women and Ethnic Studies, 935 Gravier St., Suite 1140, New Orleans, LA 70112, USA

**Keywords:** infant mortality, race, health inequity, social determinants

## Abstract

In the US, the non-Hispanic Black infant mortality rate exceeds the rate among non-Hispanic Whites by more than two-fold. To explore factors underlying this persistent disparity, we employed a mixed methods approach with concurrent quantitative and qualitative data collection and analysis. Eighteen women participated in interviews about their experience of infant loss. Several common themes emerged across interviews, grouped by domain: individual experiences (trauma, grieving and counseling; criminalization); negative interactions with healthcare providers and the healthcare system; and broader contextual factors. Concurrently, we estimated the Black infant mortality rate (deaths per 1000 live births) using linked live birth-infant death records from 2010 to 2013 in every metropolitan statistical area in the US. Poisson regression examined how contextual indicators of population health, socioeconomic conditions of the Black population, and features of the communities in which they live were associated with Black infant mortality and inequity in Black–White infant mortality rates across 100 metropolitan statistical areas with the highest Black infant mortality rates. We used principal components analysis to create a Birth Equity Index in order to examine the collective impact of contextual indicators on Black infant mortality and racial inequity in mortality rates. The association between the Index and Black infant mortality was stronger than any single indicator alone: in metropolitan areas with the worst social, economic, and environmental conditions, Black infant mortality rates were on average 1.24 times higher than rates in areas where conditions were better (95% CI = 1.16, 1.32). The experiences of Black women in their homes, neighborhoods, and health care centers and the contexts in which they live may individually and collectively contribute to persistent racial inequity in infant mortality.

## 1. Introduction

Decades of medical and public health research have failed to explain or reduce race-associated differences in infant mortality in the US. Non-Hispanic (NH) Black women are more than twice as likely to experience the loss of a child before age one compared to NH White women [1]. This mortality gap between NH Black and NH White infants is largely driven by higher rates of preterm birth among NH Back women and infant death due to preterm-related causes [2]. However, increased spending on health care nationally and improvements in health care quality and access have failed to reduce disparities in preterm birth or mortality, implicating the role of more upstream factors disproportionately experienced by NH Black women. 

The historical and perpetuated oppression of Blacks in the US has resulted in a society stratified by race—a social, cultural, and political classification [3]—and a system of unequal access to resources and opportunities [4,5]. Research is beginning to highlight the importance of this harmful social context (the macro-level conditions shaped by historical and contemporary policy), as a determinant of health and health inequities [6,7,8,9]. The persistence of disproportionately high Black infant mortality rates underscores the need to think more broadly about underlying causes. Previous work has examined race-associated differences in infant mortality as a result of differences in socioeconomic context (education, poverty, and income) between racial groups [10]. However, evidence of a racialized disparity even among women of higher socioeconomic positions [11] suggests chronic stress [12], racism [13], and other characteristics of the social and physical environment in which women are born, live, and work may additionally impact their reproductive health [14,15,16]. Documenting women’s individual experiences, their interpersonal interactions, and understanding their social and structural context is critical to the development of programs and policies designed to prevent infant mortality and eliminate the disproportionate experience of loss among NH Black women. 

The purpose of this analysis was to add Black women’s voices to the sparse literature on the social determinants of Black infant mortality and to identify factors associated with racial inequities in infant mortality rates in the US context. We interviewed women who self-identified as African American or Black and had suffered the loss of an infant during or shortly after birth. We additionally examined NH Black infant mortality rates in 100 US metropolitan areas with the goal of identifying community-level characteristics associated with increased risk and potentially amenable to community- or policy-based interventions to improve population health and promote health equity. 

## 2. Materials and Methods 

### 2.1. Study Background

Both the qualitative and quantitative findings presented in this mixed-methods study represent the work of the National Birth Equity Collaborative (NBEC; http://birthequity.org/) [17]. The underlying assumption of mixed methods studies is that the collection of qualitative and quantitative data allows for a more complete inquiry into complex phenomena and more rigorous inter-related analysis [18,19]. The rationale for using mixed methods in this study was to gather more comprehensive data while employing a research design where quantitative and qualitative data were collected concurrently. By giving qualitative and quantitative data equal priority in the data analysis, the impact of social context could be systematically examined in the investigation of factors that contribute to the disproportionately high rates of Black infant mortality. Because both were completed simultaneously, findings were triangulated so that each informed the other as analyses were conducted (e.g., measures or proxies of measures, where they existed quantitatively, for themes discussed in qualitative interviews were explored for inclusion in the index; close examination of qualitative themes were re-examined based on key quantitative measures that were significantly linked to mortality). Institutional Review Boards approved the qualitative and quantitative components prior to the conduct of research. 

Women who shared their personal experiences of loss were identified through professional relationships in active sites of the Campaign for Black Babies, an initiative by NBEC that seeks to reduce Black infant mortality through research, parent-centered collaboration, and advocacy. Quantitative explorations of contextual factors associated with NH Black infant mortality included data on all Black infant deaths occurring in the top 100 Metropolitan Statistical Areas (MSAs) ranked by Black infant mortality rate, which includes all 7 Campaign for Black Babies cities (Baltimore, Chicago, Cleveland, Detroit, Memphis, New Orleans, and Jackson). 

### 2.2. Qualitative Data Collection 

The goal of the qualitative data collection was to interview women who experienced the loss of an infant either through stillbirth, through complications of preterm delivery, or in the neonatal period. Eighteen women were identified through contacts with NBEC partner organizations including local health departments, medical centers, and non-profit organizations. Women were selected by staff of NBEC partner organizations for their previous participation in Fetal and Infant Mortality Review (FIMR), their willingness to participate, and upon their evaluation on a pre-screening document. The pre-screening document measured for the following eligibility criteria: over 18 years of age, self-identifying as African American or Black, living in their Campaign city through the gestation and birth of their child, not having clinical psychological diagnosis, not having any current substance abuse diagnosis or undergoing substance abuse treatment. The results of the pre-screening document were evaluated and shared with NBEC staff before confirming the interview. 

Eighteen interviews were conducted in summer and fall of 2016, typically lasted 1–3 h, and occurred in a private location that was most convenient to the participant (their home or office, for example). All participants consented to have their interview recorded and transcribed, and were compensated US $100 for their travel expenses, childcare and/or time lost from work. Semi-structured interviews were conducted with participants to understand their interactions with health providers, the circumstances of their child’s death, and their lived experiences prior to becoming pregnant. The interview guide was informed by NBEC staff and partners and approved by the Institutional Review Board.

### 2.3. Qualitative Data Analysis 

The interviews were recorded, transcribed verbatim, and analyzed by two independent researchers using thematic content analysis. Open coding was used to identify themes within the transcript to create the initial coding framework from data generated in each interview. The researchers independently reviewed the initial coding framework for duplications to develop a shorter list of categories informed by the analytical and theoretical grounding of the study. The sentiment (positive or negative) expressed by each woman in discussing a particular theme was also tracked for frequency. Reviewer’s cross referenced each other’s work after manual coding. We applied a social-ecologic framework to group narrow themes more broadly by level of the social ecological model to form overarching themes of individual experiences, interpersonal interactions, and community factors. 

### 2.4. Quantitative Data Collection 

The National Center for Health Statistics (NCHS) provided annual linked birth/infant death files from 2010 to 2013, inclusive, including geographic identifiers (Federal Information Processing Standard codes [FIPS]) for county of maternal residence. In some states, incomplete linkage of all infant deaths to their corresponding birth record results in a small proportion of unlinked infant deaths. Therefore, a weight is added to correct in part for biases in the percent of records linked by major characteristic. Accounting for weighted data, we computed the 4-year infant mortality rate (IMR, deaths per 1000 live births) among NH Black women in US metropolitan statistical areas (MSA). While NH Black infant mortality was our primary outcome of interest, we additionally computed rate ratios (NH Black IMR/NH White IMR) and absolute rate differences (NH Black IMR-NH White IMR) between NH Black and NH White women in each MSA in order to identify determinants of racial inequity in infant mortality. For ease of presentation, all remaining references to racial subgroups are implicitly non-Hispanic when referred to with terms Black or White. 

A comprehensive set of IMR contextual indicators characterizing the top 100 MSAs with the highest Black infant mortality rates (listed in Supplemental Table S1) were compiled and evaluated for associations with IMR among Black infants and with racial inequity in IMRs (Table 1). Selection of indicators was guided by data availability at the county or MSA-level, a theoretical foundation for and exploratory evidence of an association with infant mortality [14], as well as themes that emerged from the qualitative interviews. Indicators selected broadly define health and opportunities for better health within the social and physical environment of each community, and the distribution of health-promoting resources among Black members of the community. These data were compiled from the U.S. Census and American Community Survey, the Bureau of Justice Statistics, the USDA’s Food Environment Atlas, the Centers for Disease Control and Prevention (CDC) Behavioral Risk Factor Surveillance System (BRFSS), and the WONDER Mortality and Environmental data. 

### 2.5. Quantitative Data Analysis

Descriptive statistics described the distribution of Black IMR and Black/White inequity in IMRs as well as all contextual indicators across the 100 MSAs. Poisson regression with robust standard errors estimated the IMR rate ratio and 95% confidence interval among Blacks associated with an interquartile range (IQR) increase in each indicator (Model A). IQR scaling is a common practice when modeling continuous exposures as it provides a more attractive intuitive interpretation of the resulting beta coefficient as opposed to contrasting per a “1-unit” increase in the indicator value. Coefficients for an IQR increase contrast the infant mortality rate in MSAs with typically “high” values of the indicator (those in the middle of the upper half of the indicator distribution) to MSAs with typically “low” values of the indicator (those in the middle of the lower half of the indicator distribution. Linear regression estimated associations between an IQR increase indicators and Black/White IMR rate ratios (Model B) and rate differences (Model C). All models accounted for observations clustered within MSA and were adjusted for the total poverty level of each MSA. 

Many of the indicators examined co-occur at harmful levels within the same context. Principal components analysis (PCA) is a useful methodology for data reduction purposes in instances where many potentially collinear variables can be combined into a smaller number of principal components. In addition to evaluating the independent association between each indicator and infant mortality, we used PCA to create an index that would capture the total variance in Black IMR explained collectively by these indicators. We used PCA to identify components, or independent orthogonal linear combinations of the indicator variables, and the amount of variance represented by each component [20]. Components with variance (eigenvalues) greater than 1 were extracted the proportion of variance contributed is summed to estimate the total proportion of variance captured by the components. Standardized scoring coefficients for each indicator acted weights, representing the relative importance of the given indicator to the overall component. Standardized values of the indicator variable from each jurisdiction were multiplied by the output scoring coefficient and summed to create a total component score. Finally, total component scores for all components retained were summed to create an index (standardized with mean 0 and standard deviation 2), with higher values representing worse health conditions. We refer to this as the Birth Equity Index. As with the individual predictors, the Birth Equity Index was rescaled to the IQR. All analyses were conducted in SAS v. 9.4 (SAS, Cary, NC, USA). 

## 3. Results

### 3.1. Qualitative Results: Descriptive Analysis of Interview Data 

Of the 18 women interviewed, most (*n* = 10) cohabitated with their partners, 11 had at least a high school diploma and 10 were employed full-time. Only three women described themselves as financially comfortable, and most received financial assistance while pregnant (10 Women, Infants and Children (WIC), 12 Supplemental Nutrition Assistance Program (SNAP). 

Eight of the women experienced a stillbirth, and other women had infants who died within a few hours to 6 weeks after birth. Eleven women gave birth prior to 37 weeks gestation (preterm). Deaths occurred several months up to 4 years prior to the interview. 

Several common themes emerged across interviews, grouped into three socioecologic domains: *individual experiences* (including trauma, grieving and counseling; criminalization); *interactions with healthcare providers and the healthcare system*; and broader *contextual factors* (including economic insecurity, transportation, housing and community, race and racism). 

### 3.2. Individual Experiences

All of the women shared histories of mental and emotional trauma throughout their lives, acknowledged its impact on their pregnancy and the additional trauma they experienced after the death of a child:

“I had the drug addict mom and moved backwards and forwards. I think that’s what brought the PTSD and depression on as I got older. And that’s the only thing I can contribute the loss of my child to, my mental state.”

“They put me on antidepressants. I just rather deal with it on my own. Some days I cry a lot. Some days I’m mad. Some days I’m OK. But, it changed me. I can say that.”

When asked what has helped them cope with the loss of their child, women talked about informal (family) and formal (grief counseling) ways of dealing with grief. Six women expressed negative sentiments about experiences accessing grief counseling:

“They [hospital staff] were like, ‘Your baby is dead. What you gonna do now?’ Basically.”

“I did counseling for a while, but I stopped. How can you sit down with somebody and they telling me how to feel? I don’t like that. They never lost a child, but tell me how to feel? I mean, we planned her. We wanted her.”

However, five women spoke about positive counseling experiences after the loss, most commonly in the form of support groups:

“I highly recommend finding a support group or focus group. That helped me tremendously like I cannot stress how deeply those people impacted me. It would really make a difference in your whole grieving process because when you go to this support group again you are surrounded by people who totally understand you because they are going through the same thing.”

“I think that if you are surrounded by people who are going through that same thing and you hear their stories and they hear your story, you come together.”

“Yeah because there were other women there too were just like me and they had stories and some things helped me out and some things helped them out. So it was good. To even share that experience with other people when we had to go through the same thing.”

### 3.3. Interactions with Healthcare Providers and the Healthcare System

All 18 women interviewed expressed at least some negative sentiments about the quality of clinical care they received and their interactions with the healthcare system. Many comments alluded to provider bias and feeling that they were receiving lower quality care because of their race, lower income, or insurance status:

“Maybe they would have given me different care. Now that I see that with certain things they treat you based on what kind of insurance you have. I didn’t know it was like that until I fell victim to it.”

“Get them in and get them out. Or make you sit there for hours before you are getting seen. Then they finally see you, they ask a few questions, they leave out the room. They’re ready to write you a prescription for something. They [the health care system] don’t care about Black people.

“So the divisiveness in my healthcare came when I had to go to the hospital and was being treated by people who I had never known, and who didn’t know anything about me.”

“I had a doctor say, ‘Oh, you’re young, you can have more kids.’ You don’t know what I have been through to even say that. Prior to my twins I had already lost two. I already had two miscarriages, two losses. I don’t want to hear that when I’m trying to fight for my child to be alive.”

“I watched documentaries, I read books. I knew what I wanted, but I was treated like I didn’t know what I wanted.”

“I remember the doctor not even looking at me. He was talking to me and he treated me like I said like a number. He said ‘How many times have you been pregnant?’ and I’m like never. Then he had to turn around like ‘Oh’ ... he’s looking like, ‘She’s just another Black girl in here and she needs health care and she probably had 4 or 5 children already.’ It’s like No! We are human. We care about our well-being just like you do.”

### 3.4. Contextual Factors

Women spoke about a number of broad, contextual features of their daily lives and the communities in which they lived that may have impacted their health and that of their child. Many faced personal financial hardships and the inability to pay for food, health care, and baby supplies, struggles they saw reflected by others in their community:

“A lot of Black people can’t afford what is necessary to make those changes, so you buy what you can afford to get cause you need to survive. And because we stay in survival mode, I feel that that is a great impact on how we’re affected in our health and other areas as well.”

In characterizing their neighborhood context, women frequently made mention of isolation and a lack of transportation to services and grocery stores:

“I didn’t have a car then. To get the faster bus, that was about a 15 min walk. So, we are in the hood. To get to the good grocery store, you would have to drive far out. Maybe 30 min away from where you stay at. It’s all city, it’s no country. It’s all city houses, buses, smog, dope boys, dope girls. It’s all city.”

“You’re walking to the bus stop and you’re carrying cans, carrying cans from the grocery store. I’m carrying those gallons of milk from the WIC. I’m on the bus, I’ve got to go downtown. Gotta get on three buses, I’m pregnant. You can’t do that.”

“But if you go to the White neighborhood you see health food stores. Yeah the good stuff, organic stuff. The Black neighborhood right now they not having those.”

Negative interactions with police and criminalization of their experience were expressed by two women:

“The police came and were asking us all types of questions like we did something wrong. They asked if she slept with us in the bed because that wouldn’t be safe and if she did, they were going to arrest us.”

“Then the policemen came, taking pictures, questioning. I went off! As if we didn’t just lose our child. They questioned us like we did something wrong. Like we are some kind of criminals.”

Finally, a majority of women felt that their race—more specifically, being Black—impacted their experience. They described how racism was a feature of their lives from interpersonal interactions to the cultural and systemic conditions in which they lived:

“I was being treated like my pregnancy was a nuisance to them where her [my coworker] pregnancy and the pregnancies of other White women were celebrated.”

“I’m a Black girl in the hood and all the people that got the power see is Black people having babies. They don’t see our conditions. They don’t see really what we go through. They don’t see the prejudices that we have to go through. A White girl 16 and pregnant and she is trash in poverty is better than a Black girl that is 16 and pregnant and she is trash in poverty.”

“There is so much more to a woman than her pregnancy. They need to understand our eating, our living situation, our support system, our mental and physical health. It all makes a difference. Look at the whole me.”

### 3.5. Quantitative Results: Associations between Contextual Indicators and Black Infant Mortality

Across 100 MSAs with the highest Black IMRs in the US, the four-year Black IMR averaged 11.7 deaths per 1000 live births (range = 5.6–16.9 per 1000), compared to 5.1 deaths per 1000 live births among Whites in the same MSAs. In every MSA, the Black IMR exceeded the White IMR (Supplemental Table S1). Relative differences between Black and White rates were greatest in Trenton, NJ; Bridgeport-Stamford-Norwalk, CT; and San Francisco-Oakland-Hayward, CA where Black infant mortality rates were more than six times higher than the rate among Whites. Absolute differences were greatest in Trenton, NJ; Wichita, KS; and Shreveport-Bossier City, LA, where Blacks experienced more than 10 excess deaths per 1000 live births compared to Whites.

Table 2 shows the mean, range, and interquartile range of each contextual indicator used in the regression modeling and Birth Equity Index. There was considerable variation in each indicator across MSAs, with the exception of air pollution levels (mean = 11.7 mg/m^3^, standard deviation (STD) = 1.5) and the number of days residents reported experiencing poor mental and physical health days (which both averaged approximately 3.5, STD = 0.5). Median household income among Whites was almost two times higher, on average, compared to median household income among Black householders.

Poisson regression estimated the magnitude of associations between contextual indicators and Black infant mortality, adjusted for poverty levels (Table 3, Model A). All of the indicators were at least marginally associated with Black IMR. Compared to MSAs in the bottom quartile of poor mental and physical health days prevalence, Black IMRs were 12–13% higher in the top quartile. MSAs where housing and community environments had some of the highest rates of homicide, racial residential segregation (isolation), limited access to healthy foods, and air pollution had 7–11% higher Black IMRs compared to MSAs where rates were in the bottom quartile. Areas where the income gap between Black and White households was large had Black IMRs that were 8% higher than areas where median household incomes were more racially equitable. Prevalences of smoking and obesity—markers of the total population health—were both strongly associated with higher Black IMR (smoking rate ratio (RR) = 1.20, 95% CI = 1.13, 1.27; obesity RR = 1.16, 95% CI = 1.10, 1.22). 

Relative racial inequity in IMR was smaller in areas with a higher prevalence of poor mental and physical health days, obesity, and smoking (Table 3, Model B). The racial gap in IMR was 15–20% larger where residential segregation was more extreme and homicide and Black unemployment rates were highest compared to areas with less segregation, lower homicide and Black unemployment rates.

Increasing segregation, air pollution, homicide and Black unemployment rates were associated with larger absolute differences in IMRs between Black and White infants (Table 3, Model C). Notably, there was on average one excess Black infant death per 1000 live births compared to Whites in the most segregated MSAs compared to the least segregated (absolute IMR rate difference = 1.14, 95% CI = 0.66, 1.62), independent of the poverty level within the MSA. 

**Figure 1:**
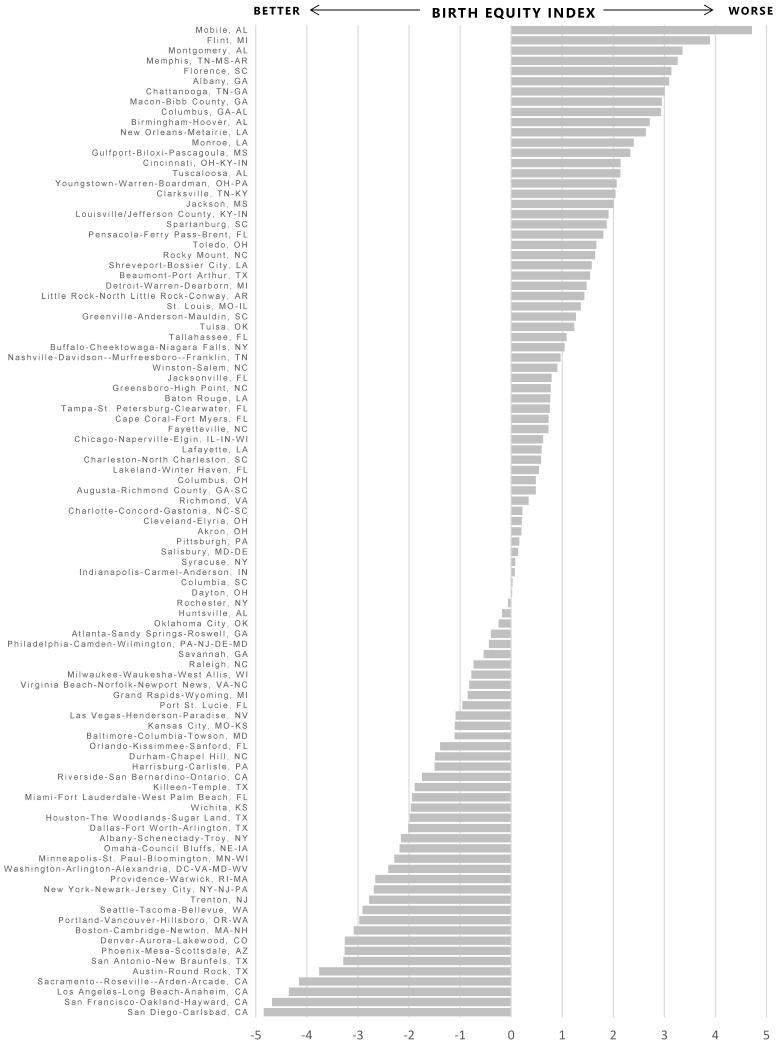
Values of the Birth Equity Index by metropolitan statistical area.

Collectively, the contextual indicators demonstrated good internal consistency reliability with Cronbach’s alpha = 0.81. The PCA analysis yielded three components to be retained with eigenvalues greater than 1 (Table 4). The three components accounted for 64% of the total variance in the data. Standardized scoring coefficients for each component were multiplied by the MSA-specific standardized values of each indicator and summed to compute the three component scores, which were summed to compute the overall Birth Equity Index value for each jurisdiction, standardized with mean 0 and standard deviation = 2. Higher values of the index indicate higher values across all of the indicators collectively, representing worse health conditions or a less healthy contextual environment relative to lower or negative values of the index. Values were highest in Mobile-AL (4.7), Flint, MI (3.9), and Montgomery, AL (3.4) MSAs and lowest in San Diego-Carlsbad, CA (−4.8), San Francisco-Oakland-Hayward, CA (−4.7), and Los Angeles-Long Beach-Anaheim, CA (−4.4) MSAs (Figure 1). Controlling for poverty levels, a 3-point (IQR) increase in the index was associated with an 24% increase in Black IMR (RR = 1.24, 95% CI = 1.16, 1.32), and a broadening of the Black/White mortality gap by almost 1 additional excess Black infant death per 1000 live births in the same jurisdiction (Black IMR to White IMR rate difference = 0.74, 95% CI = 0.23, 1.25). 

## 4. Discussion

Reducing the incidence of Black infant death is an urgent and necessary step towards achieving health equity and improving the health of the entire nation. While they represented only 21% of the births in all 20 jurisdictions from 2010 to 2013, 40% of the deaths in the same time period were Black infants. Black/White infant mortality gaps averaged nearly seven excess Black infant deaths per 1000 live births, and Black infants were more than 2.5 times more likely to die before celebrating a first birthday. 

Research on effective strategies to reduce health disparities requires systematic identification of policy-relevant contextual factors associated with Black infant mortality [21]. The stories and concerns shared by Black women who experienced the loss of an infant included their personal challenges with mental health, trauma, grieving and counseling (individual experiences), their negative interpersonal interactions with health care providers and the health care system, and the struggles they faced within their households, neighborhoods, communities, and our racially-stratified society (contextual factors). Concurrently, and informed by qualitative results, we aimed to quantify how social contextual factors impact Black infant mortality at the population level given that these were the only available contextual factors available at all geographies in the quantitative data. We examined how mental and physical health of the population (poor mental and physical health days, obesity and smoking rates), socioeconomic conditions of the Black population (educational attainment, employment, and income relative to Whites), and features of the communities in which they live (racial residential segregation, air pollution, access to healthy food, homicide, and jail admission rates) were associated individually and collectively with Black infant mortality. Apparent in both qualitative and quantitative findings was the salience of social context in shaping women’s experience of infant loss. Additionally, apparent was the entrenched and systemic racism underlying their negative interactions with health care providers, manifesting in broad socioeconomic inequity, and shaping the segregated neighborhoods in which they lived.

All quantitative indicators were at least marginally positively associated with higher Black IMR, above and beyond the detrimental effect of poverty. While two indicators considered the status of the Black population alone (Black unemployment and less than high school educational attainment), more strongly associated were the two indicators that considered the status of Blacks relative to Whites: racial income inequality and racial residential segregation. This finding is in line with growing acceptance of both as fundamental causes of health inequity [22,23]. Racial residential segregation is the physical separation of racial groups in residential contexts or the uneven spatial distribution of racial groups within a city. Racial residential segregation stems at least in part from discriminatory mortgage lending, population differences in buying power, and federal housing policies resulting in neighborhoods that are disproportionately occupied by one racial group or another [24]. Black IMRs were higher in areas characterized by greater racial residential isolation (a frequently used measure of residential segregation [25]) and wider gaps in median household incomes between Blacks and Whites. Moreover, racial residential isolation was strongly associated with racial inequity in IMRs such that the Black IMR was 20% higher than the White IMR in these areas. Previous work highlights the impact of racial residential segregation patterns on adverse birth outcomes among Black women, including preterm birth [26,27,28,29,30,31]. Further evidence suggests that the association between segregation and adverse birth outcomes may be mediated by greater exposure to violence, a psychosocial stressor with impacts on biologic health [28]. The association between homicide rates and Black IMR did not reach statistical significance in these data, but the effect may have been stronger in more local geographic areas (urban centers) where rates are higher.

Many of the indicators measured within the whole population (prevalence of smoking, obesity, limited food access, and unhealthy days) had negative beta coefficients in the racial inequity models, suggesting that higher prevalence’s were associated with smaller racial inequity in IMR. This is not indicative of a health benefit associated with these factors, but rather a result of their adverse impact on all women, Whites included. Higher prevalence’s of these indicators sharply increased White IMRs, bringing them closer to Black IMRs and thereby decreasing the racial gap between Black and White IMRs. For example, White IMRs were on average 17% higher in MSAs with the highest prevalence’s of residents with limited access—almost double the impact on Black IMRs. The contextual indicators examined are highly correlated and attributing the impact on Black IMR to a single one neglects its effect on others, and muddies interpretations [32]. Together they shape health-promoting or harming- contexts, and the utility of PCA methodology was to develop a composite index to empirically summarize the “status” of Black residents both within their own community and relative to Whites. The cumulative impact of all of the indicators considered was greater than any single indicator alone. An IQR increase in the index was associated with a 24% increase in Black IMR implying that Black IMR in jurisdictions with the worst social, economic, and environmental conditions was typically 1.24 times higher than Black IMR in jurisdictions with better social, economic and environmental conditions. Of note, the index was also associated with White IMR, with similar magnitude. The harmful context quantified by the index was not specific to Black women alone, evidence that improvements to total population health may require targeted efforts to address racial disadvantage [33]. 

These results should be considered in light of their limitations. While we aimed to triangulate results from qualitative and quantitative findings, data on some of the most salient themes emergent from the interviews is not available at MSA or lower geographic levels (e.g., perceived quality of health care, provider bias and interactions, and trauma). We did capture a set of indicators that holistically represented aspects of the social and physical environment of each MSA known to impact health, including health behaviors, socioeconomic indicators, criminal justice and crime, air pollution, and racism. This is by no means a complete listing of social determinants of Black infant mortality, but one that reliably captures a large proportion of the variability in these data. While we did consider other indicators, (e.g., measures of housing quality, food insecurity, use of public transportation and availability of physical and mental health care providers), exploratory analysis suggested that these factors had little impact on Black IMRs in these jurisdictions. While not all indicators were available in racially-stratified estimates (smoking, homicide, air pollution, for example), they capture community-wide exposures that may impact risk of infant mortality among all women, or be particularly harmful to those already made vulnerable by social marginalization. Second, given the unavailability of cohort data to identify multi-level predictors (and mediators) of mortality among Black infants, we were limited to ecologic analysis of infant mortality rates across jurisdictions. We chose to examine MSAs both in order to capture a sufficient number of infant deaths to ensure stable rate estimates as well as to achieve a more comparable unit of analyses than using estimates from a single, urban-core county which may not capture conditions in surrounding suburban counties. It may be that some indicators act on more local levels such as the neighborhood, city, or county. Estimated rate ratios compare women across MSAs, and cannot be translated to individual-level estimates of risk. As individual-level data become available, the application of hierarchical modeling may provide further detail on the magnitude of risk associated with contextual factors for Black women and the causal pathways through which exposure to harmful contexts increase the likelihood of their infant’s death. Finally, we did not include the experience of women being treated for or dealing with current substance abuse issues to lessen their risk of re-traumatization during the interview. While mental health and counseling referrals were prepared for each interview, we had limited capacity to provide on-site, medically-appropriate counseling for grieving and potentially unstable women.

## 5. Conclusions

Achieving birth equity—conditions of optimal births for all people [17]—requires addressing racial and social inequities in a sustained effort. Ongoing failure to reduce Black infant mortality and eliminate racial inequity motivated our attempt to move beyond evaluation of strictly biomedical risk factors to consider more holistically the voices, experiences, and contexts of Black women in the US. We identified a number of harmful features of the social environment disproportionately impacting Black women and undergirding the unacceptably high rates of Black infant mortality. Implementing universal interventions—those that target better health and well-being for the entire population—may effectively reduce population-level rates of infant mortality, but not without first addressing the fundamental causes of racial inequities in health [22,23]. Use of a mixed methods approach allowed us to hear directly from Black women about their experiences of infant loss while at the same time utilizing contextual data on features of the communities in which they lived to identify and quantify risk of infant death at the population level. Future studies should focus on identifying the ways in which harmful features of the context in which women live become embodied, influencing their perceptions and experiences, and physically impacting their health. Our findings suggest that eliminating racial stratification in infant mortality will require efforts aimed at improving access to resources and opportunities in the Black community and the conditions into which Black children are born and thrive.

## Figures and Tables

**Table 1:** Indicator description and data source.

Indicator	Definition	Source and Year
Education	% of Non-Hispanic (NH) Black residents age 25 and older with less than a high school education	American Community Survey, 2009–2013 5-year estimate
Unemployment	% of NH Black residents in the civilian labor force who are unemployed	American Community Survey, 2009–2013 5-year estimate
Residential segregation	Isolation index is the minority-weighted average of the minority population i neach area. Values range from 0 (complete integration) to 1 (complete segregation)	Census, 2010
Adult smoking	% of the adult population that currently smokes	Behavioral Risk Factor Surveillance System (BRFSS)
Poor mental health days	Average number of mentally unhealthy days reported in the past 30 days (age-adjusted)	BRFSS, 2006–2012 average
Poor physical health days	Average number of physically unhealthy days reported in the past 30 days (age-adjusted)	BRFSS, 2006–2012 average
Adult obesity	% of adults that report a body mass index (BMI) of ≥30	Centers for Disease Control and Prevention (CDC) Diabetes Interactive Atlas, 2011
Limited access to healthy foods	% of the population who are low-income and do not live close to a grocery store.	United States Department of Agriculture (USDA) Food Environment Atlas, 2010
Homicide rate	Homicide deaths per 100,000 residents	CDC WONDER mortality data, 2006–2012 average
Air pollution	Daily fine particulate matter (average daily measure in micrograms per cubic meter).	CDC WONDER Environmental Data, 2011
Jail admissions	Annual admissions per 100,000 residents age 15–64	Bureau of Justice Statistics, 2012
Structural racism (Racial inequality in income)	NH White to NH Black ratio of median household income	American Community Survey, 2009–2013 5-year estimate

Census and American Community Survey indicators were available at the Metropolitan Statistical Area (MSA)-level and 2013 5-year estimates were used. For the remaining indicators, county-level estimates were averaged across counties comprising a MSA. Data years for indicators are those available closest to or during the 2010–2013 time frame of infant deaths in order to preserve temporality.

**Table 2:** Descriptive statistics for 100 MSAs included in analysis.

	Mean (Standard Deviation, STD)	Min	Max	IQR *
Total IMR, deaths per 1000 live births	6.86 (1.61)	3.92	12.14	2.01
NH Black IMR, deaths per 1000 live births	11.66 (2.19)	5.62	16.94	2.55
NH White IMR, deaths per 1000 live births	5.08 (1.17)	2.32	8.44	1.62
NH Black/NH White IMR ratio	2.38 (0.61)	1.10	6.26	0.67
NH Black/NH White IMR difference	6.58 (1.89)	0.53	13.89	2.29
Social Determinants Indicators				
NH Black unemployment, %	16.84 (3.24)	10.64	27.09	4.27
NH Black population age 25 and older with less than a high school diploma, %	17.17 (4.34)	9.06	30.19	5.87
Structural racism (ratio of NH White to NH Black median household income)	1.80 (0.23)	1.20	2.35	0.30
Residential segregation (isolation)	42.97 (16.05)	8.80	80.90	21.95
Smoking prevalence among adults, %	19.77 (3.72)	10.38	26.69	5.57
Obesity prevalence among adults, %	29.82 (3.95)	18.94	36.90	5.07
Limited access to healthy foods, %	6.36 (3.09)	1.19	16.83	3.85
Homicide rate, per 100,000 population	6.23 (2.86)	2.18	15.20	3.39
Air pollution (particulate matter <2.5 micrometers in aerodynamic diameter), mg/m^3^	11.70 (1.54)	7.87	14.54	2.16
Jail admission rate, per 100,000 population	6428.23 (2598.94)	1544.3	13,033.73	3524.22
Poor mental health days, n	3.57 (0.46)	2.54	4.80	0.57
Poor physical health days, n	3.65 (0.49)	2.60	4.93	0.76
Poverty, %	15.63 (3.18)	8.20	26.10	3.65

* IQR = interquartile range.

**Table 3:** Associations between social determinants indicators and infant mortality rates in 100 MSAs, 2010–2013. *

	Model A ^a^			Model B ^b^			Model C ^c^		
Indicator	RR	95% CI		Beta	95% CI		Beta	95% CI	
NH Black unemployment	1.06	1.01	1.11	0.15	0.02	0.28	0.47	0.03	0.91
NH Black less than high school education	1.05	0.95	1.17	0.13	−0.04	0.30	0.35	−0.22	0.92
Structural racism (racial income inequality)	1.08	1.01	1.16	0.14	−0.01	0.28	0.48	−0.03	0.98
Residential segregation (isolation)	1.10	1.05	1.15	0.20	0.06	0.35	1.14	0.66	1.62
Smoking prevalence among adults	1.20	1.13	1.27	−0.26	−0.46	−0.06	0.22	−0.39	0.82
Obesity prevalence among adults	1.16	1.10	1.22	−0.20	−0.35	−0.05	0.41	−0.04	0.86
Limited access to healthy foods	1.09	1.01	1.19	−0.09	−0.21	0.03	0.01	−0.43	0.45
Homicide rate	1.07	0.99	1.15	0.19	0.03	0.34	0.57	0.08	1.06
Air pollution	1.11	1.03	1.19	−0.02	−0.14	0.10	0.56	0.13	0.99
Jail admission rate	1.06	0.96	1.18	−0.12	−0.33	0.09	−0.07	−0.82	0.69
Poor mental health days	1.12	1.04	1.20	−0.21	−0.37	−0.06	−0.25	−0.72	0.22
Poor physical health days	1.13	1.03	1.25	−0.33	−0.53	−0.13	−0.32	−0.96	0.31

* All models adjusted for poverty rate in each jurisdiction; ^a^ Model A is the rate ratio comparing NH Black infant mortality rates across an interquartile range (IQR) increase in the indicator; ^b^ Model B beta estimates represent the change in magnitude of the rate ratio comparing NH Black and NH White IMRs for an IQR increase in the indicator; ^c^ Model C beta estimates represent the change in magnitude of the rate difference between NH Black and NH White IMRs for an IQR increase in the indicator.

**Table 4:** Principal component analysis of Black IMR social determinant indicators in 100 MSAs.

	Principal Component 1	Principal Component 2	Principal Component 3
Eigenvalue	4.41	2.02	1.20
% of total variance explained	36.7%	16.8%	10.0%
Indicator Variable Loadings			
NH Black unemployment, %	−0.43	0.68	0.20
NH Black less than high school education, %	0.38	0.53	−0.16
Structural racism (racial income inequality)	−0.04	0.70	−0.39
Residential segregation (isolation)	0.44	0.60	−0.08
Smoking prevalence among adults, %	0.38	0.29	0.49
Obesity prevalence among adults, %	0.74	0.19	0.14
Limited access to healthy foods, %	0.64	−0.14	0.21
Homicide rate, per 100,000	0.79	0.06	−0.02
Air pollution, mg/m^3^	−0.04	0.66	0.35
Jail admission rate, per 100,000	0.77	−0.23	0.04
Poor mental health days, *n*	−0.04	−0.06	0.90
Poor physical health days, *n*	0.25	−0.02	0.79

## Supplementary Materials

Supplementary File 1

## References

[1] Matthews T.J., MacDorman M.F., Thoma M.E. (2015). Infant mortality statistics from the 2013 period linked birth/infant death data set. Natl. Vital Stat. Rep..

[2] Lorenz J.M., Ananth C.V., Polin R.A.D., Alton M.E. (2016). Infant mortality in the United States. J. Perinatol..

[3] Marks J. (2005). New information, enduring questions: Race, genetics, and medicine in the 21st century. Genewatch.

[4] Feagin J., Bennefield Z. (2014). Systemic racism and U.S. health care. Soc. Sci. Med..

[5] Krieger N. (2012). Methods for the scientific study of discrimination and health: An ecosocial approach. Am. J. Public Health.

[6] Do D.P., Finch B.K., Basurto-Davila R., Bird C., Escarce J., Lurie N. (2008). Does place explain racial health disparities? Quantifying the contribution of residential context to the Black/White health gap in the United States. Soc. Sci. Med..

[7] Subramanian S.V., Jones K., Kaddour A., Krieger N. (2009). Revisiting Robinson: The perils of individualistic and ecologic fallacy. Int. J. Epidemiol..

[8] Lukachko A., Hatzenbuehler M.L., Keyes K.M. (2014). Structural racism and myocardial infarction in the United States. Soc. Sci. Med..

[9] Siddiqi A., Jones M.K., Bruce D.J., Erwin P.C. (2016). Do racial inequities in infant mortality correspond to variations in societal conditions? A study of state-level income inequality in the USA, 1992–2007. Soc. Sci. Med..

[10] Fry-Johnson Y.W., Levine R., Rowley D., Agboto V., Rust G. (2010). United States Black: White infant mortality disparities are not inevitable: Identification of community resilience independent of socioeconomic status. Ethn. Dis..

[11] Schoendorf K.C., Hogue C.J., Kleinman J.C., Rowley D. (1992). Mortality among infants of Black as compared with White college-educated parents. N. Engl. J. Med..

[12] Kramer M.R., Hogue C.J., Dunlop A.L., Menon R. (2011). Preconceptional stress and racial disparities in preterm birth: An overview. Acta Obstet. Gynecol. Scand..

[13] Hogue C.J., Hargraves M.A. (1993). Class, race, and infant mortality in the United States. Am. J. Public Health.

[14] Kim D., Saada A. (2013). The social determinants of infant mortality and birth outcomes in western developed nations: A cross-country systematic review. Int. J. Environ. Res. Public Health.

[15] World Health Organization (2008). Closing the Gap in a Generation: Health Equity through Action on the Social Determinants of Health. http://apps.who.int/iris/bitstream/10665/43943/1/9789241563703_eng.pdf.

[16] Collins J.W., Hawkes E.K. (1997). Racial differences in post-neonatal mortality in Chicago: What risk factors explain the Black infant’s disadvantage?. Ethn. Health.

[17] National Birth Equity Collaborative 2016. http://birthequity.org/.

[18] Tashakkori A.M., Teddlie C.B. (2010). Handbook of Mixed Methods in the Social and Behavioral Sciences.

[19] Creswell J.W. (2014). Research design: Qualitative, Quantitative, and Mixed Methods Approaches.

[20] Everitt B.S., Dunn G. (1993). Principal components analysis. Applied Multivariate Data Analysis.

[21] Williams D.R., Mohammed S.A. (2013). Racism and health ii: A needed research agenda for effective interventions. Am. Behav. Sci..

[22] Williams D.R., Collins C. (2001). Racial residential segregation: A fundamental cause of racial disparities in health. Public Health Rep..

[23] Marmot M. (2005). Social determinants of health inequalities. Lancet.

[24] Rothwell J.T., Massey D.S. (2010). Density zoning and class segregation in U.S. metropolitan areas. Soc. Sci. Q..

[25] US Census Bureau Racial and Ethnic Segregation in the United States: 1980–2000. https://www.census.gov/hhes/www/housing/resseg/pdf/app_b.pdf.

[26] Britton M.L., Shin H. (2013). Metropolitan residential segregation and very preterm birth among African American and Mexican-origin women. Soc. Sci. Med..

[27] Debbink M.P., Bader M.D. (2011). Racial residential segregation and low birth weight in Michigan’s metropolitan areas. Am. J. Public Health.

[28] Kramer M.R., Cooper H.L., Drews-Botsch C.D., Waller L.A., Hogue C.R. (2010). Metropolitan isolation segregation and Black-White disparities in very preterm birth: A test of mediating pathways and variance explained. Soc. Sci. Med..

[29] Mason S.M., Messer L.C., Laraia B.A., Mendola P. (2009). Segregation and preterm birth: The effects of neighborhood racial composition in North Carolina. Health Place.

[30] Nyarko K.A., Wehby G.L. (2012). Residential segregation and the health of African-American infants: Does the effect vary by prevalence?. Matern. Child Health J..

[31] Grady S.C. (2006). Racial disparities in low birthweight and the contribution of residential segregation: A multilevel analysis. Soc. Sci. Med..

[32] Messer L.C., Laraia B.A., Kaufman J.S., Eyster J., Holzman C., Culhane J., Elo I., Burke J.G., O’Campo P. (2006). The development of a standardized neighborhood deprivation index. J. Urban Health.

[33] Cerda M., Tracy M., Ahern J., Galea S. (2014). Addressing population health and health inequalities: The role of fundamental causes. Am. J. Public Health.

