# Supplementary material for: “Look at the Whole Me”: A Mixed-Methods Examination of Black Infant Mortality in the US through Women’s Lived Experiences and Community Context
Source: Int J Environ Res Public Health. 2017 Jul 5;14(7):727. doi: 10.3390/ijerph14070727 (PMC5551165; doi:10.3390/ijerph14070727)
Supplement: Supplementary File 1 [file ijerph-14-00727-s001.docx]

**Supplemental Table 1.**  Total and race-specific infant mortality rates (IMR) in 100 US metropolitan areas, 2010-2013.

| **Metropolitan Area** | **IMR** | **Black IMR** | **White IMR** |
| --- | --- | --- | --- |
| Akron, OH | 7.01 | 10.84 | 5.96 |
| Albany, GA | 11.97 | 15.33 | 7.93 |
| Albany-Schenectady-Troy, NY | 6.42 | 12.49 | 4.92 |
| Atlanta-Sandy Springs-Roswell, GA | 5.81 | 8.29 | 4.34 |
| Augusta-Richmond County, GA-SC | 7.89 | 11.30 | 5.45 |
| Austin-Round Rock, TX | 4.54 | 10.07 | 3.66 |
| Baltimore-Columbia-Towson, MD | 6.78 | 12.04 | 3.75 |
| Baton Rouge, LA | 9.18 | 13.15 | 6.32 |
| Beaumont-Port Arthur, TX | 7.14 | 11.90 | 6.55 |
| Birmingham-Hoover, AL | 9.33 | 15.37 | 6.90 |
| Boston-Cambridge-Newton, MA-NH | 3.92 | 6.73 | 3.16 |
| Bridgeport-Stamford-Norwalk, CT | 4.26 | 9.35 | 2.32 |
| Buffalo-Cheektowaga-Niagara Falls, NY | 7.79 | 14.16 | 6.06 |
| Cape Coral-Fort Myers, FL | 5.92 | 12.61 | 3.86 |
| Charleston-North Charleston, SC | 5.87 | 10.58 | 3.89 |
| Charlotte-Concord-Gastonia, NC-SC | 6.14 | 10.88 | 4.41 |
| Chattanooga, TN-GA | 7.22 | 11.97 | 6.18 |
| Chicago-Naperville-Elgin, IL-IN-WI | 6.37 | 12.76 | 4.34 |
| Cincinnati, OH-KY-IN | 7.99 | 14.66 | 6.49 |
| Clarksville, TN-KY | 6.32 | 12.56 | 4.21 |
| Cleveland-Elyria, OH | 7.43 | 13.90 | 4.83 |
| Columbia, SC | 7.21 | 10.88 | 4.99 |
| Columbus, GA-AL | 9.06 | 13.17 | 5.14 |
| Columbus, OH | 7.72 | 12.81 | 6.36 |
| Dallas-Fort Worth-Arlington, TX | 6.42 | 11.34 | 5.13 |
| Dayton, OH | 7.64 | 15.64 | 5.73 |
| Denver-Aurora-Lakewood, CO | 5.19 | 10.49 | 3.87 |
| Detroit-Warren-Dearborn, MI | 7.73 | 13.26 | 5.50 |
| Durham-Chapel Hill, NC | 6.01 | 12.15 | 4.25 |
| Fayetteville, NC | 7.59 | 11.61 | 5.95 |
| Flint, MI | 7.63 | 12.77 | 5.69 |
| Florence, SC | 12.14 | 15.85 | 8.44 |
| Grand Rapids-Wyoming, MI | 5.82 | 12.58 | 4.89 |
| Greensboro-High Point, NC | 8.42 | 12.37 | 6.41 |
| Greenville-Anderson-Mauldin, SC | 6.86 | 11.40 | 5.53 |
| Gulfport-Biloxi-Pascagoula, MS | 8.11 | 12.62 | 7.21 |
| Harrisburg-Carlisle, PA | 6.07 | 12.13 | 4.42 |
| Hartford-West Hartford-East Hartford, CT | 5.45 | 11.20 | 3.83 |
| Houston-The Woodlands-Sugar Land, TX | 5.68 | 10.33 | 4.79 |
| Huntsville, AL | 7.08 | 10.99 | 5.50 |
| Indianapolis-Carmel-Anderson, IN | 7.34 | 11.64 | 6.33 |
| Jackson, MS | 9.24 | 12.88 | 4.95 |
| Jacksonville, FL | 7.21 | 11.93 | 5.07 |
| Kansas City, MO-KS | 5.59 | 10.40 | 4.51 |
| Killeen-Temple, TX | 7.73 | 12.93 | 6.28 |
| Lafayette, LA | 7.42 | 11.32 | 5.68 |
| Lakeland-Winter Haven, FL | 7.19 | 9.20 | 6.59 |
| Las Vegas-Henderson-Paradise, NV | 5.06 | 9.61 | 4.41 |
| Little Rock-North Little Rock-Conway, AR | 7.35 | 11.69 | 5.71 |
| Los Angeles-Long Beach-Anaheim, CA | 4.24 | 8.52 | 3.12 |
| Louisville/Jefferson County, KY-IN | 6.32 | 10.12 | 5.53 |
| Macon-Bibb County, GA | 9.88 | 12.99 | 6.46 |
| Memphis, TN-MS-AR | 9.31 | 12.62 | 5.35 |
| Miami-Fort Lauderdale-West Palm Beach, FL | 5.02 | 8.99 | 3.67 |
| Milwaukee-Waukesha-West Allis, WI | 7.45 | 14.50 | 4.76 |
| Minneapolis-St. Paul-Bloomington, MN-WI | 4.61 | 8.36 | 3.89 |
| Mobile, AL | 9.12 | 12.74 | 6.74 |
| Monroe, LA | 7.83 | 9.06 | 6.87 |
| Montgomery, AL | 8.47 | 11.51 | 6.22 |
| Nashville-Davidson--Murfreesboro--Franklin, TN | 6.13 | 10.49 | 5.40 |
| New Haven-Milford, CT | 5.27 | 9.65 | 3.99 |
| New Orleans-Metairie, LA | 7.13 | 10.33 | 4.95 |
| New York-Newark-Jersey City, NY-NJ-PA | 4.24 | 8.26 | 2.65 |
| Oklahoma City, OK | 6.95 | 11.55 | 6.52 |
| Omaha-Council Bluffs, NE-IA | 5.24 | 10.78 | 4.38 |
| Orlando-Kissimmee-Sanford, FL | 6.93 | 12.46 | 5.02 |
| Pensacola-Ferry Pass-Brent, FL | 7.40 | 12.24 | 5.86 |
| Philadelphia-Camden-Wilmington, PA-NJ-DE-MD | 7.44 | 12.89 | 4.38 |
| Phoenix-Mesa-Scottsdale, AZ | 5.58 | 11.14 | 4.75 |
| Pittsburgh, PA | 6.22 | 12.50 | 5.15 |
| Portland-Vancouver-Hillsboro, OR-WA | 4.78 | 7.55 | 4.56 |
| Port St. Lucie, FL | 5.40 | 9.85 | 3.96 |
| Providence-Warwick, RI-MA | 5.73 | 9.54 | 4.57 |
| Raleigh, NC | 6.25 | 11.83 | 4.54 |
| Richmond, VA | 7.67 | 13.42 | 4.11 |
| Riverside-San Bernardino-Ontario, CA | 5.50 | 10.26 | 5.20 |
| Rochester, NY | 6.28 | 11.97 | 4.86 |
| Rocky Mount, NC | 7.94 | 11.36 | 3.92 |
| Sacramento--Roseville--Arden-Arcade, CA | 4.80 | 9.42 | 3.85 |
| St. Louis, MO-IL | 6.39 | 12.40 | 4.34 |
| Salisbury, MD-DE | 7.02 | 14.19 | 5.14 |
| San Antonio-New Braunfels, TX | 5.84 | 9.47 | 4.51 |
| San Diego-Carlsbad, CA | 4.17 | 6.92 | 3.42 |
| San Francisco-Oakland-Hayward, CA | 3.92 | 9.17 | 2.77 |
| Savannah, GA | 6.64 | 11.44 | 4.94 |
| Seattle-Tacoma-Bellevue, WA | 4.32 | 7.83 | 3.83 |
| Shreveport-Bossier City, LA | 10.31 | 15.97 | 5.68 |
| Spartanburg, SC | 4.98 | 5.62 | 5.09 |
| Syracuse, NY | 5.50 | 9.81 | 4.55 |
| Tallahassee, FL | 8.35 | 12.75 | 5.12 |
| Tampa-St. Petersburg-Clearwater, FL | 7.03 | 13.01 | 5.34 |
| Toledo, OH | 7.37 | 11.77 | 6.15 |
| Trenton, NJ | 6.92 | 16.53 | 2.64 |
| Tulsa, OK | 7.34 | 11.96 | 6.54 |
| Tuscaloosa, AL | 9.54 | 14.31 | 5.96 |
| Virginia Beach-Norfolk-Newport News, VA-NC | 8.37 | 13.17 | 5.56 |
| Washington-Arlington-Alexandria, DC-VA-MD-WV | 5.69 | 9.72 | 3.65 |
| Wichita, KS | 7.26 | 16.94 | 6.15 |
| Winston-Salem, NC | 8.36 | 15.18 | 6.65 |
| Youngstown-Warren-Boardman, OH-PA | 7.94 | 14.88 | 6.55 |
